# Stage-Specific Changes in the Water, Na^+^, Cl^-^ and K^+^ Contents of Organelles during Apoptosis, Demonstrated by a Targeted Cryo Correlative Analytical Approach

**DOI:** 10.1371/journal.pone.0148727

**Published:** 2016-02-11

**Authors:** Frédérique Nolin, Jean Michel, Laurence Wortham, Pavel Tchelidze, Vincent Banchet, Nathalie Lalun, Christine Terryn, Dominique Ploton

**Affiliations:** 1 Laboratoire de Recherche en Nanosciences, EA 4682, Université de Reims Champagne Ardenne, Reims, France; 2 CNRS UMR 7369, Université de Reims Champagne Ardenne, Reims, France; 3 Platform of Cellular and Tissular Imaging (PICT), Université de Reims Champagne Ardenne, Reims, France; Laval University Cancer Research Centre, CANADA

## Abstract

Many studies have demonstrated changes in the levels of several ions during apoptosis, but a few recent studies have reported conflicting results concerning the changes in water content in apoptotic cells. We used a correlative light and cryo-scanning transmission electron microscopy method to quantify water and ion/element contents simultaneously at a nanoscale resolution in the various compartments of cells, from the onset to the end of apoptosis. We used stably transfected HeLa cells producing H2B-GFP to identify the stages of apoptosis in cells and for a targeted elemental analysis within condensed chromatin, nucleoplasm, mitochondria and the cytosol. We found that the compartments of apoptotic cells contained, on average, 10% more water than control cells. During mitochondrial outer membrane permeabilization, we observed a strong increase in the Na^+^ and Cl^-^ contents of the mitochondria and a strong decrease in mitochondrial K^+^ content. During the first step in apoptotic volume decrease (AVD), Na^+^ and Cl^-^ levels decreased in all cell compartments, but remained higher than those in control cells. Conversely, during the second step of AVD, Na^+^ and Cl^-^ levels increased considerably in the nucleus and mitochondria. During these two steps of AVD, K^+^ content decreased steadily in all cell compartments. We also determined *in vivo* ion status during caspase-3 activity and chromatin condensation. Finally, we found that actinomycin D-tolerant cells had water and K^+^ contents similar to those of cells entering apoptosis but lower Na^+^ and Cl^-^ contents than both cells entering apoptosis and control cells.

## Introduction

Cell shrinking, also known as apoptotic volume decrease (AVD), is a structural hallmark of apoptosis [1]. It has been clearly demonstrated that cell death is not initiated by shrinkage, but more likely by the fluxes of several ions (Na^+^, K^+^ and Cl^-^ in particular) [2]. These fluxes modify the concentrations of ions, for which specific levels are required for both the initiation and progression of apoptosis. Ion fluxes may also generate a loss of water leading to cell shrinking [3].

Cell physiology studies have shown that most of the water molecules within organelles are involved in hydrating the macromolecules and are essential, together with ions, for their folding and activity [4–6]. The most recent methods for quantifying cell volume and water content during apoptosis have been limited to the study of entire individual cells, without a more detailed analysis of their organelles [3, 7]. Moreover, as these methods have produced divergent results [8], hydration of apoptotic cells is still an open field. We recently developed a correlative light and cryo-scanning transmission electron microscopy (cryo-STEM) method [9, 10] for the simultaneous quantification of water and ions at the nanoscale, within cell compartments. We used this method to study the changes in water and ion contents in the various organelles of cancerous cells during apoptosis induced with actinomycin D (AMD). We used stably transfected HeLa cells producing histone H2B tagged with GFP (H2B-GFP) to identify the stages of apoptosis. This clear identification of stages was necessary because apoptosis proceeds within the different cells of a cell culture at different times after the addition of the apoptosis-inducing drug [11–13], and because new data concerning the onset of apoptosis are urgently required [14]. We first studied the timing of successive stages identified on the basis of the shape of nuclei and chromatin condensation, by time-lapse imaging and GFP fluorescence studies. We then correlated these stages with mitochondrial depolarization, cytochrome-*c* diffusion, caspase-3 and PARP activation. Finally, we applied our correlative light and cryo-STEM method to ultrathin sections of cell populations during the course of apoptosis. All the cells present in the ultrathin cryo-sections were classified relatively to stages of apoptosis on the basis of H2B-GFP fluorescence and we then quantified water and various elements and ions (N, P, S, K^+^, Cl^-^, Mg^2+^ and Na^+^) at the nanoscale within the various cell compartments.

## Materials and Methods

### Cell culture

HeLa cells stably expressing H2B-GFP (provided by K. Monier, University of Lyon, France) were cultured in DMEM (Gibco) supplemented with 10% fetal bovine serum in 25cm^2^ Nunc flasks, with passaging twice weekly (at confluence). All cultures tested negative for mycoplasma infection.

### Time-lapse imaging

For the imaging of cells dynamics during apoptosis induced by 500 ng/mL AMD, HeLa cells stably expressing histone H2B tagged with GFP (H2B-GFP) were used to seed Ø21 mm uncoated glass-bottomed “Ibidi μ-Dish-500” Petri dishes (Ibidi GmbH, Germany). To study mitochondrial depolarization during apoptosis [15], we added tetramethylrhodamine ethyl ester (TMRE) at a final concentration of 40 nM and incubated the cells for 30 minutes before imaging. After adding 500 ng/mL AMD, dishes were placed on the stage of an LSM 710-NLO laser scanning confocal microscope (Zeiss Microsystems, Gennevilliers, France) enclosed in an XL-5 dark LS 2000 incubator (PeCon, Germany) maintained at 37°C with a heating unit and a temperature controller. For three-dimensional time-lapse imaging (4D and 5D), images were acquired with a 63x Plan-apochromat 1.4 NA oil objective (Zeiss Microsystems, Gennevilliers, France). Two-photon excitation at 860 nm, with a CHAMELEON femtosecond titanium-sapphire laser (Coherent, Santa Clara, CA) at a power of 1.5% was used to simultaneously generate GFP and TMRE fluorescence with limited phototoxicity and photobleaching [16]. Differential interference contrast (DIC) imaging was also performed simultaneously with a specific detector for transmitted light, to investigate changes in the morphology of the cells and their main compartments (organelles in the cytoplasm, nuclei and nucleoli).

Typically, *z*-stacks of 512 x 512 pixels (corresponding to a field of view of 134.69 μm x 134.69 μm) containing 62 to 85 slices were simultaneously acquired in three channels (GFP fluorescence, TMRE fluorescence and DIC), every 5 minutes for 7 h 15. In these conditions, 62 to 85 *z*-stacks were collected with an *xyzt* resolution of 0.26 x 0.26 x 0.3 μm x 5 minutes for each experiment. Each *z*-stack was then processed with Amira® 5.6 (FEI VSG, Mérignac, France) and Imaris® (Bitplane, Zurich, Switzerland) to perform 3D visualization of GFP and TMRE fluorescence at each time point. For this, we used two procedures. The first one, called surface rendering, computes a solid 3 D surface of an object boundary by using only pixels of the latter which are defined by a fixed grey level. The second one, called volume rendering, computes a transparent view of an object by using the different grey levels of all its voxels. To analyze the changes of nuclear volume and of TMRE intensity in each chosen cell, we performed a semi-quantitative analysis of GFP and TMRE signals. First, we used Imaris software to quantify the volume of all voxels containing H2B-GFP fluorescence which is approximately the volume of the nucleus. Second, we used ImageJ software (NIH, Bethesda) to quantify TMRE intensity. We defined a region of interest (ROI) surrounding a cell of interest. We then sum the intensity of all pixels containing TMRE fluorescence in this ROI in all optical sections of each z-stack (on a 32 bits scale). We calculated the mean pixel intensity of TMRE fluorescence in this ROI for each time point. Finally, as we evidenced that TMRE outside of mitochondria produced a faint fluorescence which disappeared after the first hour of the experiment, we decided to analyze the changes of TMRE intensity relatively to its value reached at time 1 hour.

We selected one optical section from each DIC *z*-stack for investigation of the morphological changes occurring within the whole cell or within the cytoplasm or the nucleus during AMD treatment. The 62 to 85 images for each channel (3D visualization of GFP fluorescence, of TMRE fluorescence and of DIC optical sections) were then assembled with Quick-time® to create movies showing the 3D changes in chromatin and in mitochondria over the course of the whole experiment.

### Immunolabeling of cytochrome c, cleaved PARP and activated caspase 3

Immunolabeling was carried out on HeLa cells stably expressing histone H2B tagged with GFP (H2B-GFP) used to seed coverslips in control conditions, in cells stressed by treatment with 50 ng/mL AMD for 3 h or cells in which apoptosis was induced by treatment with 500 ng/mL AMD for 7 h. Cells were simultaneously fixed and permeabilized with 4% paraformaldehyde and 0.1% Triton-X100 (Sigma, Saint Quentin Fallavier, France) for 5 minutes at room temperature. Non-specific binding sites were saturated by incubation for 30 minutes with 3% BSA (cytochrome *c*, cleaved PARP) or 10% normal goat serum (activated caspase 3). Cells were labelled by incubation for 30 minutes at room temperature with monoclonal mouse anti-cytochrome *c* (clone 6H2.B4 which specifically recognizes the native form of human cytochrome *c*) diluted 1:20.000 (Novus Biological), anti-cleaved PARP (1:50) (Dako, Trappes, France), mouse anti- activated caspase 3 (1:50) (Santa Cruz Biotechnology, Tebu-Bio, Le Perray en Yvelines, France) (Sigma) antibodies. Biotinylated (1:50) (Jackson, Interchim, Montluçon, France) or Alexa Fluor-coupled secondary antibodies (1:50) (Molecular Probes, Life Technologies, Saint Aubin, France) were then incubated with the cells for 30 minutes. Streptavidin-Alexa-Fluor complexes (Molecular Probes) were added and the mixture was incubated for 30 minutes. The Alexa Fluor labels used were Alexa 568 (1:2000) and Alexa 647 (1:800). Coverslips were mounted in Citifluor.

*Z*-stacks of 40 to 60 optical sections, with 512 x 512 pixels and 2 ^16^ gray levels, were acquired with an LSM 710 confocal microscope (Zeiss Microsystems, Gennevilliers, France) equipped with a 63x Plan-apochromat 1.4 NA oil objective (Zeiss Microsystems, Gennevilliers, France). GFP, Alexa 568 and Alexa 647 were excited either sequentially or simultaneously with different laser lines: 488 nm (argon laser); 561 nm (laser diode) and 633 nm (HeNe laser). Differential interference contrast (DIC) imaging was simultaneously performed with a specific detector for transmitted light, to investigate the morphological features of the cells and their main compartments (organelles in the cytoplasm, nuclei and nucleoli). Each *z*-stack was then processed with Amira® 5.6 (FEI, Mérignac, France) to obtain a surface or a volume rendering image for one, two or three fluorescent markers.

### Targeted nano-analysis of water and ions in cell compartments by cryo-correlative microscopy

We quantified water and elements/ions with our recently developed cryo-correlative microscopic approach coupling fluorescence imaging for the identification of H2B-GFP-tagged chromatin and scanning transmission electron microscopy (STEM) imaging. STEM imaging was used for the nano-quantification of water by dark-field imaging and of elements by energy dispersive X-ray spectroscopy [9, 10, 17]**.** A detailed description of our method can be found in [17], and we provide a brief description here. HeLa cells stably expressing histone H2B tagged with GFP (H2B-GFP) were used to seed 25 cm^2^ Nunc flasks at a density of 100 000 cells/mL. Cells in control conditions and cells in which apoptosis was induced by treatment with 500 ng/mL AMD for 7 h were analyzed.

Cells were pelleted by centrifugation and then cryofixed by rapid plunging into liquid ethane with no cryoprotectant or chemical fixation. Vitreous 80 nm-thick sections were cut and collected on formvar-carbon-coated London finder grids (Agar Scientific), which were directly transferred to a GATAN 626 EM cryo-holder that was then inserted into a custom-built cryostage for fluorescence imaging. This cryostage allowed us to maintain cryosections at– 171°C for 1.5 h and to record high-quality images with a Zeiss Axioscope Vario A1 equipped with a 50x objective (0.55 NA; 9.1 mm working distance). Nuclei of interest were then imaged and localized relative to letters, numbers and two central triangles marked on the grid. The cryo-holder was then transferred into a CM 30 (FEI) or a JEOL 2100 FEG STEM (JEOL Company) electron microscope and the cryo sections were freeze-dried. The grid was placed in exactly the same position as in the light microscope, by imaging the two central triangles of the grid and adjusting their positions with the translation and rotation functions of the scanning transmission electron microscope. After this adjustment, the nuclei identified by fluorescence imaging were easily located by STEM imaging. Images were then recorded for the measurement of water content and for the identification of cell compartments in which elements were quantified by EDX spectrometry. Cell compartments were identified on the basis of two parameters: their nuclear fluorescence status and their cytoplasmic morphology. Within the nucleus, condensed chromatin is highly fluorescent whereas the surrounding nucleoplasm is not fluorescent. Identification of the condensed and uncondensed chromatin was improved by applying a RGB rainbow LUT to fluorescence images (S1 Fig). In the cytoplasm, the morphology of mitochondria was easy to identify due to their well preserved cristae, whereas areas outside the mitochondria and containing no identifiable structures were identified as cytosol. The clear identification of these four compartments facilitated the drawing of regions of interest (ROIs) for energy dispersive X-ray spectrometry. Thus, elements such as nitrogen (N), phosphorus (P) and sulfur (S) were quantified, together with ions such as the sodium (Na^+^), magnesium (Mg^2+^), chloride (Cl^-^) and potassium (K^+^) ions and their concentrations were calculated in mmol/L, making use of the known water contents of the ROIs investigated.

All analyses were performed on three different cultures for each set of conditions. As the duration of some stages of apoptosis was extremely short, the probability of detecting them was low. In these conditions, for each stage, we analyzed a total of three to eighty-three different cells. The data shown are means ± SEM from triplicate analyses.

## Results

### Time-lapse imaging of cells following the induction of apoptosis by actinomycin D

**Fig 1:**
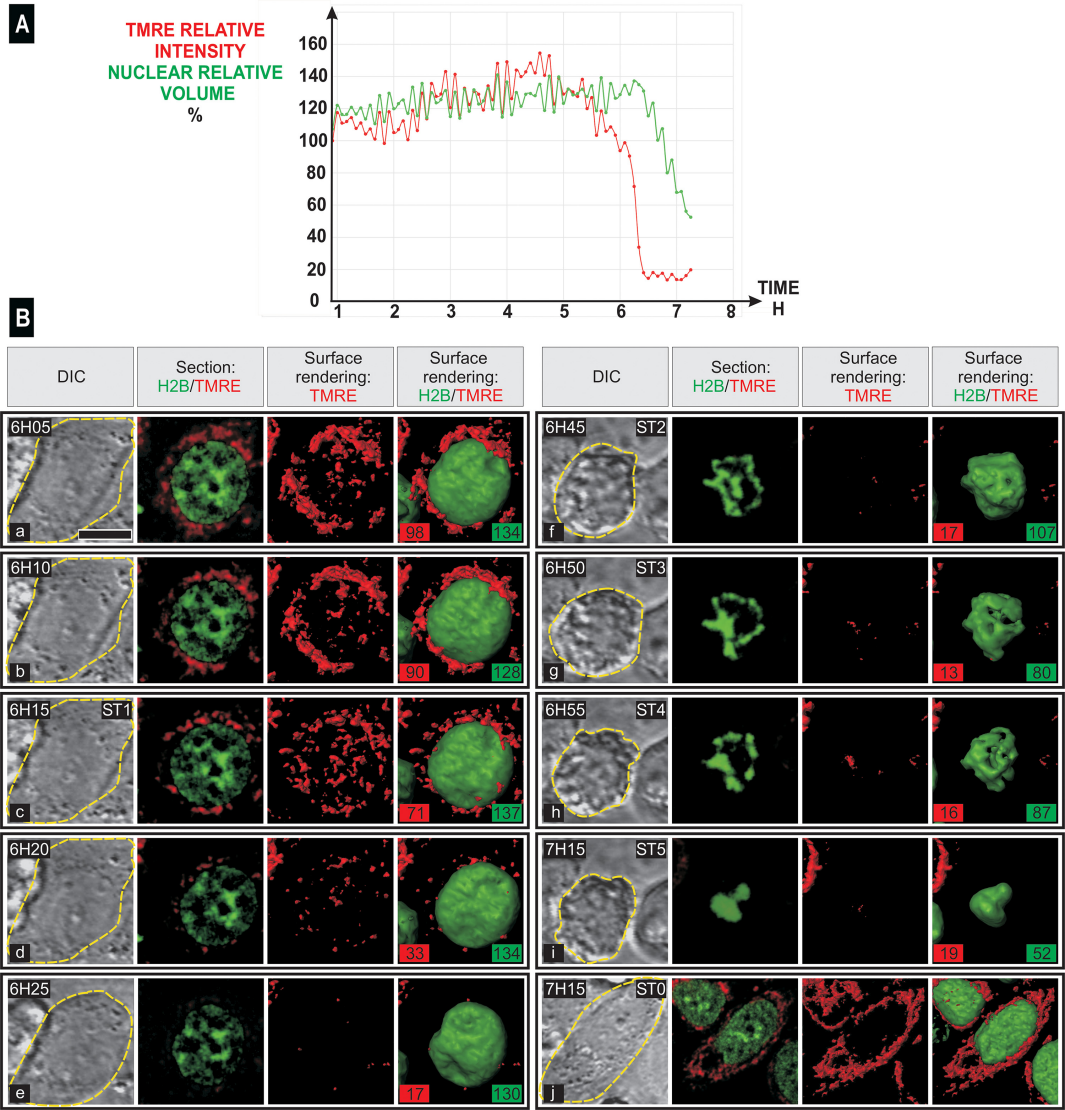
Simultaneous imaging of cell shape, mitochondrial potential and nuclear modifications at the onset and during the various stages of apoptosis. HeLa cells stably expressing H2B-GFP were stained with TMRE to study mitochondrial polarization. Simultaneous time-lapse confocal imaging of cell shape (DIC), TMRE and H2B-GFP was performed by two-photon excitation every five minutes for 7 hours and 15 minutes after the induction of apoptosis by the addition of 500 ng/mL AMD. (A) Traces for TMRE intensity (red line, relative to value reached at time 0.91 h) and nuclear volume (green line, relative to value at time 0 h) in a representative cell (cell #9 on S1–S3 Movies). Mitochondrial depolarization began at 6 h 05 minutes and ended at 6 h 25 minutes, when nuclear volume began to decrease. (B) Cell shape, 3D structure of the TMRE signal, chromatin and nucleus. For each time point, one DIC image (left), one optical section for the red and green signals, a 3D view (surface rendering) of the TMRE signal and a 3D view (surface rendering) of both TMRE signal (red) and H2B-GFP (green) are shown. On DIC image, yellow dotted line indicates the limit of the cell. On 3D view of both TMRE signal (red) and H2B-GFP (green), the relative intensity of the red signal and the volume of the nucleus are indicated by the red and green labels, respectively. Typical chromatin and nucleus structures defined the main stages of apoptosis: stage 1 (ST 1) to stage 5 (ST 5). At the far right of the bottom row, one cell unaffected by AMD after 7 h and 15 minutes is defined as a stage 0 cell (ST 0). In this cell, TMRE staining appears as a 3D network of filaments and the angular nucleus contains a segregated nucleolus. The scale bar represents 10 μm.

We investigated the timing of nuclear modifications relative to the loss of mitochondrial potential [18] during apoptosis induced by actinomycin D (AMD, 500 ng/mL) in HeLa H2B-GFP cells loaded with TMRE. We recorded Z-stacks of optical sections by two-photon excitation every five minutes, for 7.15 hours after the addition of AMD for the simultaneous imaging of DIC contrast, of green (H2B-GFP) and red (TMRE) signals. After 3D processing of data recorded in many different fields of view and of different cell cultures, we found that 30 to 60% of cells shrunk between 3.45 and 6.30 h after the addition of AMD (S1 and S2 Movies and S1 Table). In each single cell engaged in apoptosis, we quantified both the relative intensity of the TMRE signal in mitochondria and the relative nuclear volume (Fig 1). The relative intensity of TMRE signal (on Fig 1A, the representative cell shown is cell #9 on S1 and S2 Movies) suddenly decreased, and this signal disappeared over a period of 15 to 20 min (between 6 h 10 minutes and 6 h 25 minutes after the addition of AMD). The relative volume of the nucleus decreased suddenly 10 minutes later. Three-dimensional imaging (see Fig 1B for the same cell and S3 Movie) showed that active mitochondria constituted a 3D network of filaments. At the 6 h 5 minutes and 6 h 10 minutes time points, the red signal formed filaments and clumps (the relative intensity of the red signal and the volume of the nucleus are indicated by the red and green labels, respectively). The red signal was organised exclusively into numerous dots at time 6 h 15 minutes and only rare tiny red dots were visible at time 6 h 20 minutes. These dots had disappeared by 6 h 25 minutes. The nucleus was largest at 6 h 15 minutes, displaying large irregular clumps of condensed chromatin, whereas the shape of the cell was unchanged as imaged in DIC contrast (indicated by the dotted line). We refer to this stage as “stage 1” (ST 1). At 6 h 25 minutes, DIC imaging evidenced the beginning of cell shrinking and budding of the plasma membrane. There was a faint red signal and several depressions were visible in the nucleus. At 6 h and 45 minutes, DIC imaging evidenced a strong shrinking of the cell and its nucleus had irregular contours, with elongated clumps of condensed chromatin located both at the periphery and in the central part of the nucleus. We refer to this stage as “stage 2” (ST 2). At 6 h and 50 minutes, corresponding to “stage 3” (ST 3), we observed condensed chromatin strictly located at the periphery of the nucleus constituting a discontinuous layer and ovoid clumps. At 6 h and 55 minutes, corresponding to “stage 4” (ST 4), condensed chromatin appeared as peripheral round clumps and at 7h 15 minutes, corresponding to “stage 5” (ST 5), the condensed chromatin formed a few large roundish masses. From ST 2 to ST 5, the volume of the nucleus decreases steadily, from 95% to 38% the volume of the nucleus at ST 1. At 7 h 15 minutes, some cells not engaged in apoptosis (last row, right-handed column in Fig 1B) were spread and characterized with a 3D filamentous mitochondrial network and with an elongated and angular nucleus containing segregated nucleoli and clumps of condensed chromatin (defined as “stage 0” (ST 0)). To study the behaviour of the latter cells, we performed two different experiments. In the first one, we maintained treatment with actinomycin D (500 ng/mL) during 15h and 17 minutes and we performed time-lapse imaging as described above to study when cells entered apoptosis (S4 Movie). As evidenced, all cells died during this long treatment. However, it is interesting to note that one unaffected cell, with a normal network of active mitochondria, was still present at time 13h53. It suddenly shrunk at time 14h00 and evidenced a strong reorganization of mitochondria and a faint TMRE staining. In a second experiment, time-lapse imaging was performed during a treatment with actinomycin D (500 ng/mL) during 7h. Then, actinomycin D was washed out and time-lapse imaging was performed during the next 16 hours and 30 minutes. Then cells were maintained in culture medium without actinomycin D during additional 72 hours and imaged again during 6h30. This experiment (S5 Movie) demonstrates that some cells continue to engage in apoptosis after wash out of actinomycin D treatment. However, at that time, several cells were not engaged in apoptosis and recovered 3 days later as evidenced by presence of numerous mitotic cells.

### Localization of cytochrome-*c*, activated caspase-3 and cleaved PARP

We then used fixed cells to determine when cytochrome *c* (Cc) release, caspase-3 activation and PARP cleavage took place relative to the different nuclear stages described above [19, 20].

#### 3D localization of Cc (Fig 2)

Anti-cytochrome-*c* antibody binding was imaged on fixed HeLa cells stably expressing H2B-GFP after the induction of apoptosis by 500 ng/mL AMD, for 7h and 15 minutes.

**Fig 2:**
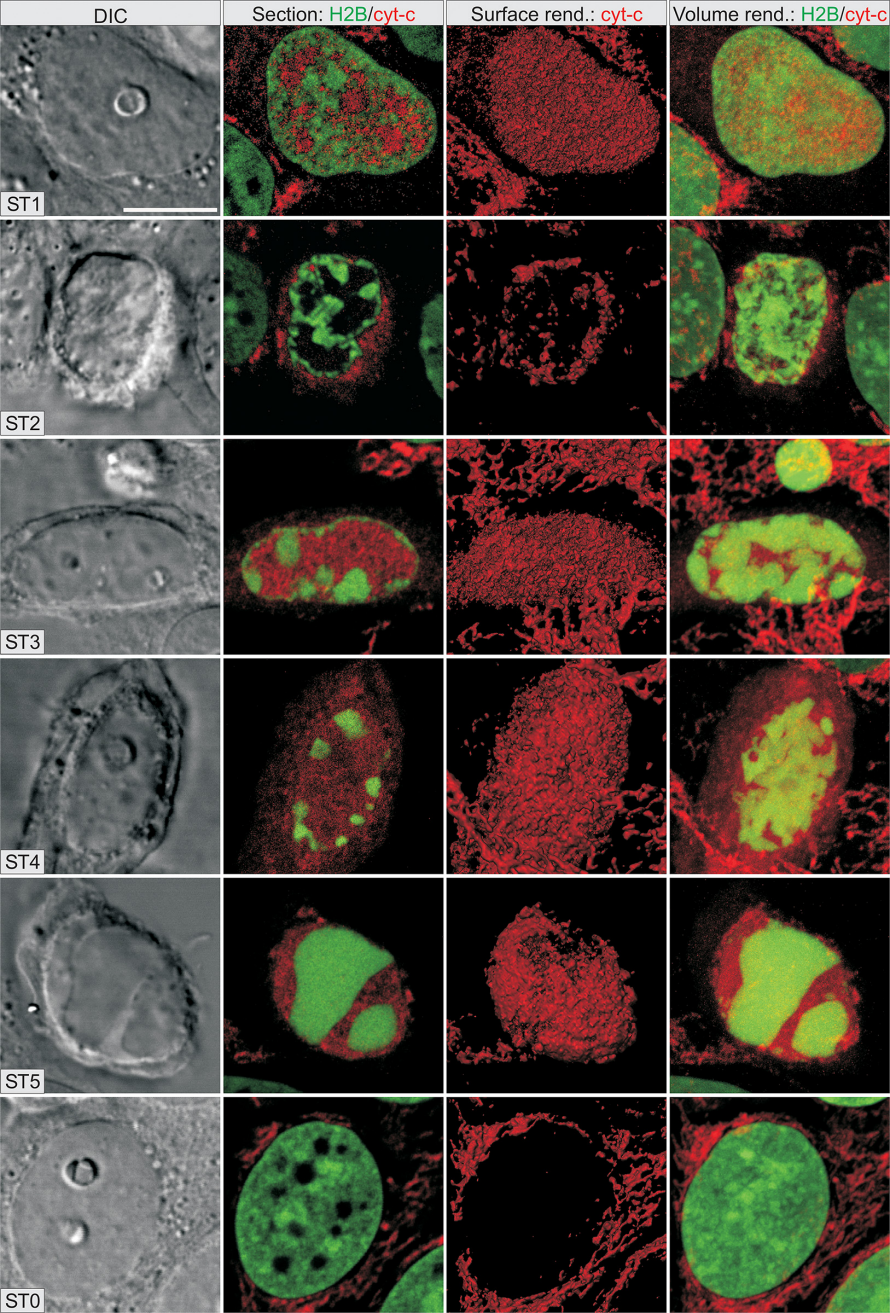
Simultaneous 3D localization of cytochrome-*c* (Cc) and H2B-GFP showing Cc redistribution during specific stages of apoptosis. Anti-cytochrome-*c* antibody binding was imaged on fixed HeLa cells stably expressing H2B-GFP after the induction of apoptosis by 500 ng/mL AMD, for 7h and 15 minutes. Four images are shown for the same cell, at a given stage. On the first image, differential interference contrast (DIC) shows the shape of the cell, nucleus and nucleolus. The second image is an optical section passing through the middle of the nucleus showing the merge of images for Cc (red) and H2B-GFP (green). The third image is a 3D surface rendering of Cc alone (red). The last image is a simultaneous 3D transparent volume rendering of both Cc (red) and H2B-GFP (green). The scale bar represents 10 μm.

In control and ST 0 cells, Cc was labeled as a network of cytoplasmic filaments typical of mitochondria. In ST 1 cells, Cc labeling was diffuse and faint in the cytoplasm and abundant in the nuclei, excluding condensed chromatin. Cc labeling took the form of aggregates dispersed throughout the cytoplasm at ST 2 to ST 5. However, Cc labelling was absent from the nucleus in ST 2 cells but present in the nuclei of cells at ST 3 to ST 5 excluding condensed chromatin. The relative 3D distribution of Cc labelling and of chromatin appear more clearly on rotation of 3D reconstructions of cells in each stage (S6 Movie).

#### 3D localization of activated caspase-3 and cleaved PARP (S2 Fig)

Anti-activated caspase-3 and anti-cleaved PARP antibodies were used to label fixed HeLa cells stably expressing H2B-GFP after the induction of apoptosis by incubation with 500 ng/mL AMD for 7 h 15 minutes. Activated caspase-3 and cleaved PARP (actCasp3 and clePARP) were absent from the cytoplasm and the nucleus of control, ST 1 and ST 0 cells. From ST 2 to ST 5, actCasp3 was detected as aggregates dispersed throughout the cytoplasm and in the nucleus, whereas clePARP was present exclusively in the nucleus. The relative 3D distribution of actCasp3, of clePARP and of chromatin appear more clearly on rotation of 3D reconstructions (S7 Movie).

**Fig 3:**
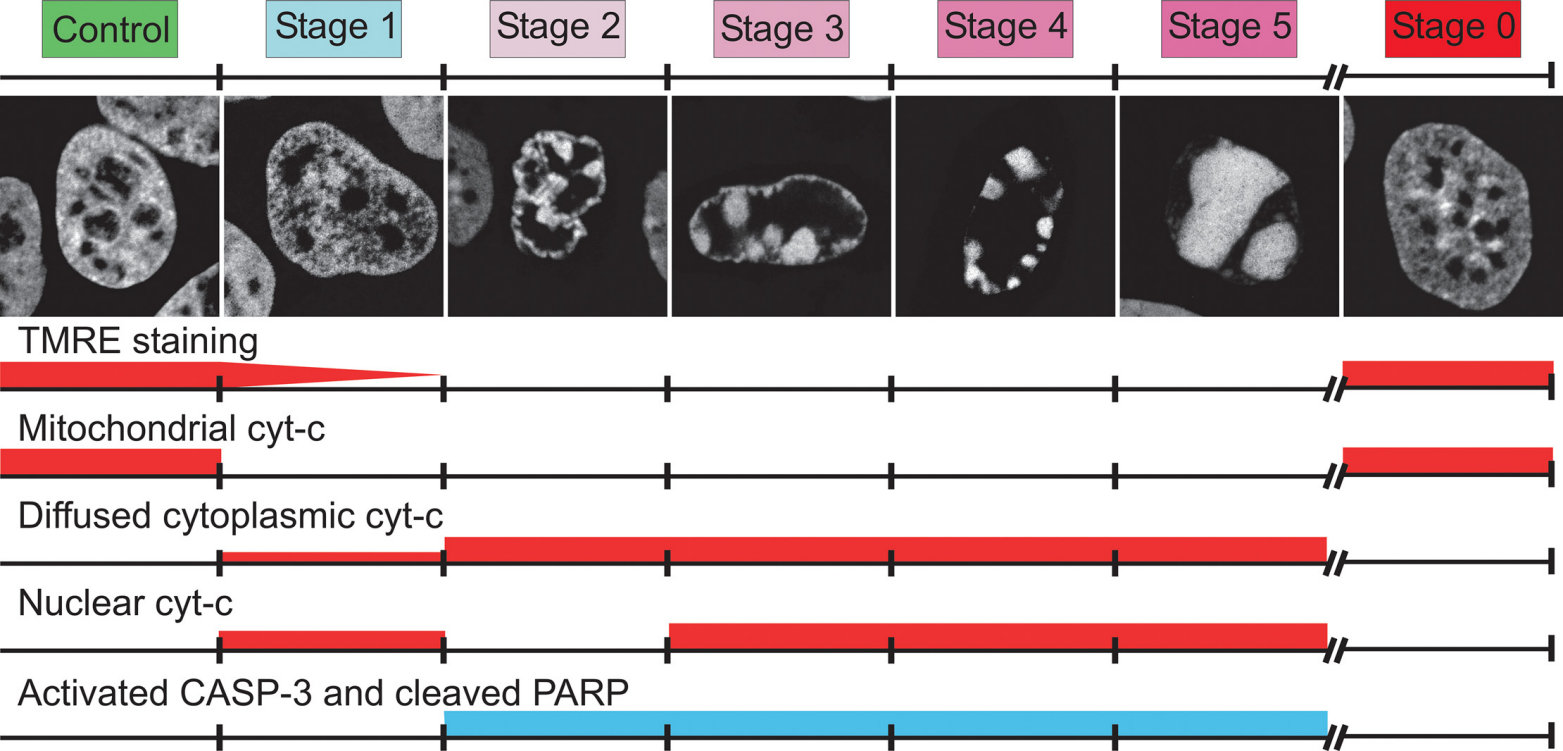
Nuclear shape and the condensation and disposition of chromatin were sufficient to identify cells i) entering apoptosis (ST 1), ii) in the course of apoptosis (ST 2 to ST 5) and iii) not engaged in apoptosis (ST 0). We confirmed this identification by TMRE staining, the localization of cytochrome-*c* (in the mitochondria, cytosol or nucleus) and the presence of activated CASP-3 and cleaved PARP.

In summary (Fig 3), all these results demonstrate that the shape of the nucleus and chromatin condensation and disposition can be used to identify cells: i) entering into apoptosis (ST 1 cells), ii) in the course of apoptosis (ST 2 to ST 5 cells) and iii) not engaged in apoptosis (ST 0 cells).

### Quantification of water and elements/ions by cryo nano-analysis

**Fig 4:**
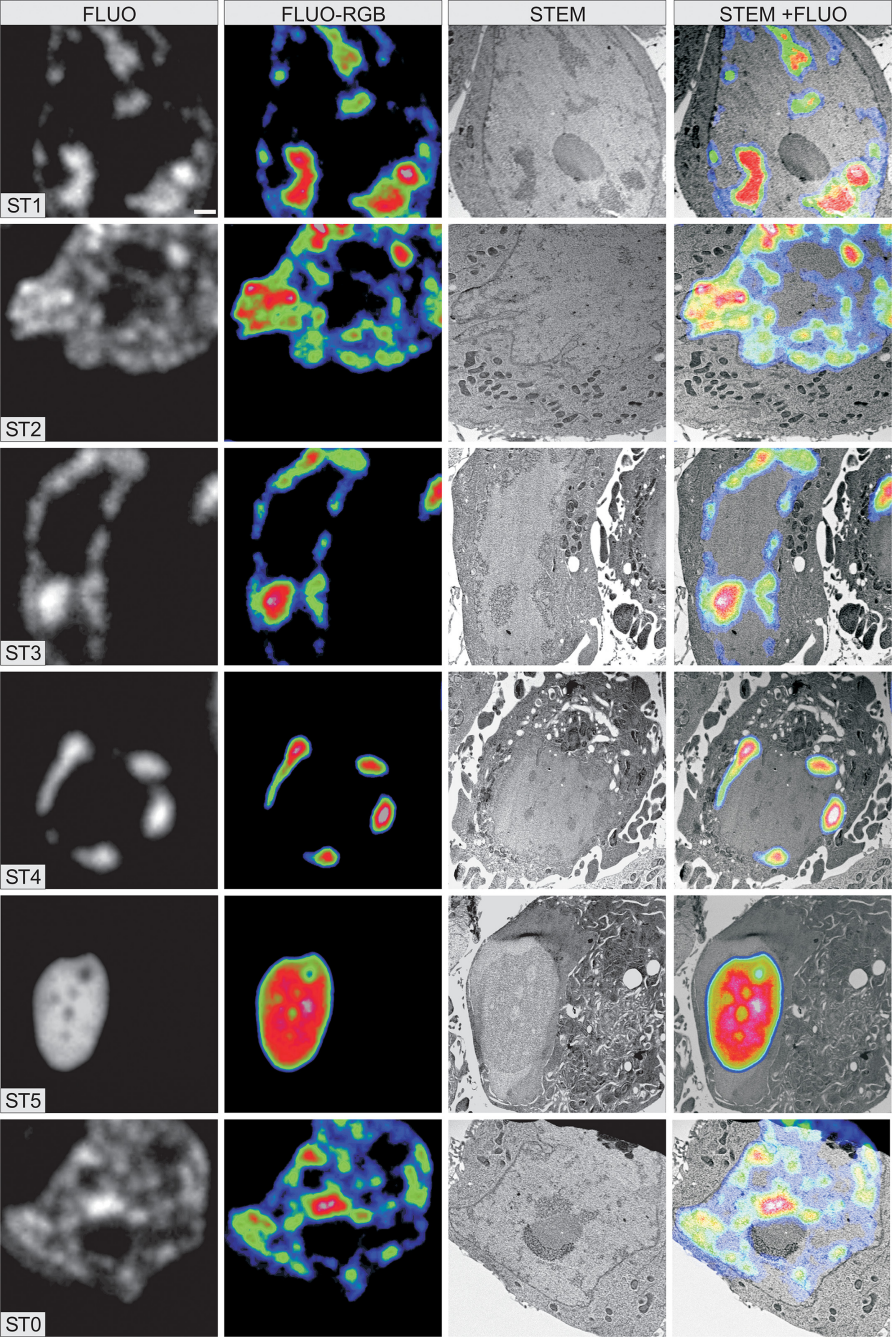
Cryo-correlative fluorescence and scanning transmission electron microscopy (STEM) of cells in the different stages of apoptosis. For each stage, four images of the same cell from a single 80 nm-thick ultrathin cryo section are shown. Fluorescence imaging of H2B-GFP in the ultrathin cryo sections (first and second panels showing fluorescence intensity on a gray scale, and after application of a rainbow RGB look-up table, respectively) made it possible to analyze chromatin distribution and nucleus shape, to identify each stage of apoptosis. The cells characterized by fluorescence imaging were then imaged by STEM (third panel). Due to a strong natural electron contrast and good ultrastructural preservation, the compartments in the cytoplasm are easily identified. Merging of the fluorescence and STEM images recorded at the same magnification (last panel) is required for a clear identification of chromatin clumps before the targeted elemental analysis of condensed chromatin and nucleoplasm. The scale bar represents 1 μm.

We then investigated changes in the water and element/ion contents of the cells during the various stages of apoptosis, with our cryo-correlative imaging technique [9, 10, 17]. We treated cells with AMD for 7h to induce apoptosis and we then cryofixed the cells directly in liquid ethane. We used H2B-GFP fluorescence on ultrathin cryosections to characterize the shape of the nuclei and the type of chromatin condensation specific to each of the stages of apoptosis (Fig 4, 1^st^ and 2^nd^ columns). We then recorded a dark-field image of the same cells by cryo-STEM (Fig 4, 3^rd^ column) to quantify dry mass and water content of cell compartments. Identification of the mitochondria and the cytosol surrounding them was easy. However, to identify nuclear compartments on the STEM image, it was necessary to merge this image with the fluorescence image. On this composite image, (Fig 4, 4^th^ column), clumps of condensed chromatin were identified with a high fluorescence whereas nucleoplasm contained no fluorescence.

**Fig 5:**
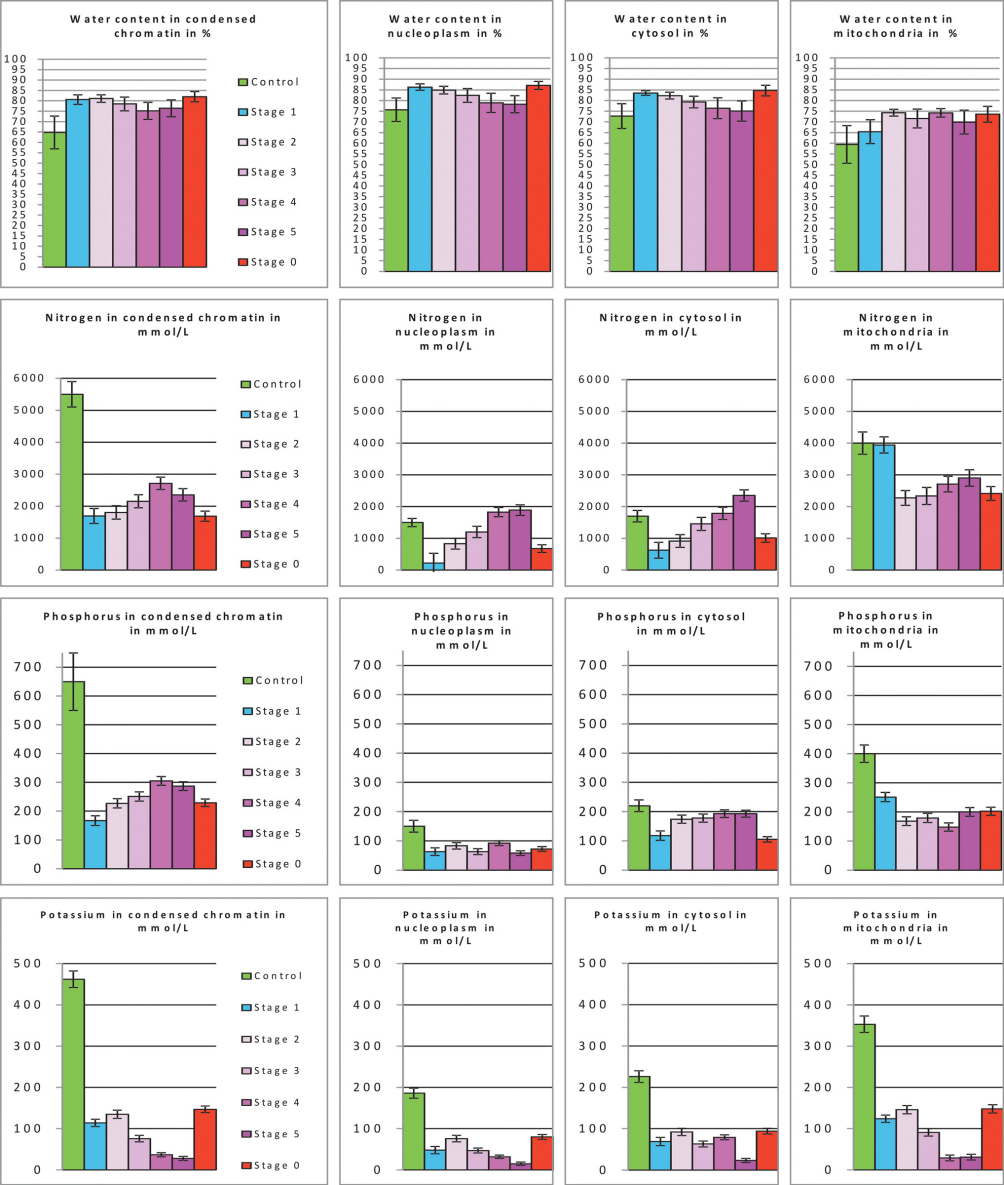
Targeted quantification of water and of N, P and K^+^ in the cytosol, mitochondria, condensed chromatin and nucleoplasm. Water percentage was calculated by STEM imaging and the concentration of element/ions (N, P and K^+^) was calculated by energy dispersive X-ray spectrometry in the cytosol, mitochondria, condensed chromatin and nucleoplasm of each of the following: i) control cells, ii) cells in various stages of apoptosis (ST 1 to ST 5 stages) and iii) cells in the ST 0 stage. Results, in mmol/L, are given as means ±SEM (*n* = 3; 3 to 83 different cells per stage).

We found that the water content of the nuclear compartments and cytosol increased by 10 to 15% between ST 1 and ST 5 (Fig 5, first line), whereas that of the mitochondria increased by 15% from ST 2 to ST 5. The water content of all cell compartments in ST 0 cells was similar to that of ST 1 cells. We then calculated a hydration index (or HI): the ratio of water content to dry matter content [10]. In all compartments of ST 1 to ST 5 cells and of ST 0 cells, HI was about twice that in control cells.

We then performed a targeted energy dispersive X-ray analysis (EDX analysis) in regions of interest in these cell compartments. We simultaneously quantified the principal ions and elements (N, P, K^+^, Na^+^, Cl^-^; S and Mg^2+^), calculating their content in mmol/L.

Detailed quantitative data for nitrogen, phosphorus and potassium are presented in Fig 5. [N] in the nucleus and cytosol of ST1 cells decreased to levels one sixth to one third those in control cells. It then increased by 50% in the nucleus and cytosol of the cells between ST 2 and ST 5. In mitochondria, [N] decreased by 50% between ST 2 and ST 5 cells. In ST 0 cells, nuclear and cytosolic [N] was similar to that of ST 1 cells and the [N] of mitochondria was half that of control cells.

[P] in all cell compartments of ST 1 cells was lower, by a factor of two to three, than that in control cells. The [P] of chromatin increased by 50% from ST 2 to ST 5, whereas that of the other compartments remained constant. In ST 0 cells, the cytosolic and mitochondrial [P] was half that of control cells.

In all compartments of control cells, [K^+^] was between 180 and 460 mmol/L. It decreased by a factor of four in all compartments of ST 1 cells and it decreased steadily between ST 2 and ST 5, eventually reaching 15 mmol/L. In all compartments of ST 0 cells, [K^+^] was similar to that of ST 1 cells.

**Fig 6:**
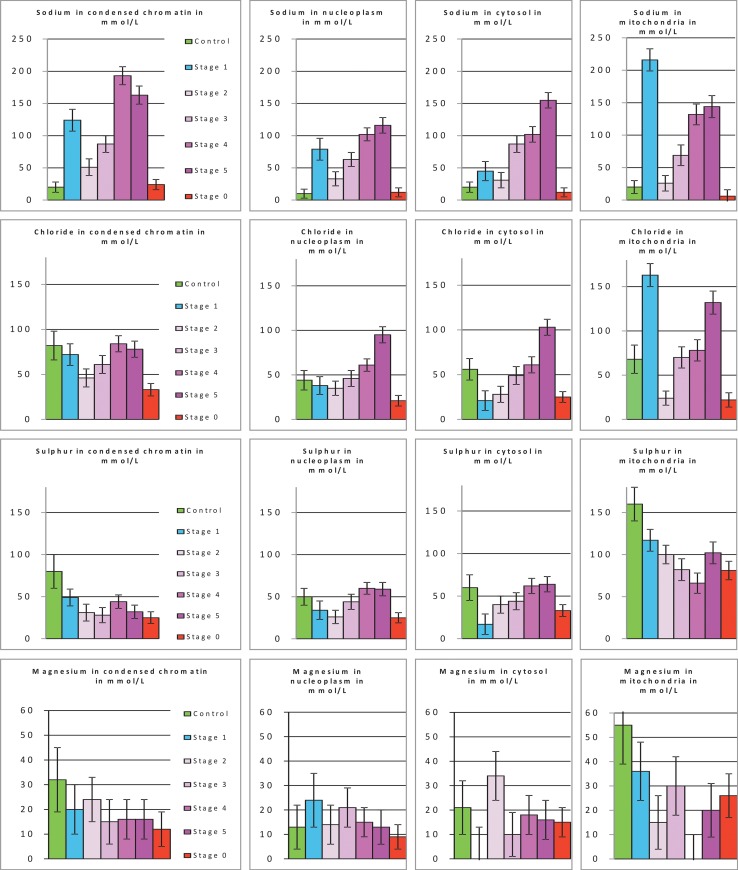
Targeted quantification of Na^+^, Cl^-^, S and Mg^2+^ in the cytosol, mitochondria, condensed chromatin and nucleoplasm. Concentration of elements/ions (Na^+^, Cl^-^, S and Mg^2+^) were determined by energy dispersive X-ray spectrometry in the cytosol, mitochondria, condensed chromatin and nucleoplasm of each of the following: i) control cells, ii) cells in the various stages of apoptosis (ST 1 to ST 5) and iii) cells in the ST 0 stage. Results, in mmol/L, are given as means ±SEM. (*n* = 3; 3 to 83 different cells per stage).

Detailed quantitative data for sodium, chloride, sulphur and magnesium are presented in Fig 6. In ST1 cells, relatively to control cells, [Na^+^] was six to eight times higher in the nuclei (80 to 120 mmol/L), two times higher in the cytosol (45 mmol/L) and ten times higher in the mitochondria (200 mmol/L). In ST 2 cells, [Na^+^] decreased to 20 to 50 mmol/L in all compartments. It then increased steadily from ST 3 to ST 5, to reach 100 to 200 mmol/L. In all compartments of ST 0 cells, [Na^+^] was similar to that of control cells. [Cl^-^] remained constant in the nucleus of ST 1 cells (40 to 80 mmol/L) but decreased by a factor of three in the cytosol and by a factor of two in the mitochondria. In ST 2 cells, [Cl^-^] remained constant, except in mitochondria, in which it decreased by a factor of seven relative to the concentration recorded in ST1 cells. Between ST 3 and ST 5 cells, [Cl^-^] increased steadily, reaching levels similar to those in control cells in the chromatin and twice those of control cells in the nucleoplasm, cytosol and mitochondria. In all compartments of ST 0 cells, [Cl^-^] was one half to one third that in control cells.

[S] decreased in all compartments of ST 1 cells. From ST 2 to ST 5, it decreased steadily in condensed chromatin and mitochondria, whereas it increased in the nucleoplasm and cytosol. In all compartments of ST 0 cells, [S] was about half that of control cells.

[Mg^2+^] differed between stages, but was very low in the cytosol of ST 1 cells and in the mitochondria of ST 4 cells.

### Ultrastructural morphology of mitochondria

During the elemental analysis, we investigated the shape of mitochondria and the structure of their cristae (S3 Fig). In control (Ctrl) and ST 0 cells, the mitochondria were elongated and had straight or slightly curved cristae. In ST 1 and ST 2 cells, some of the mitochondria were even more elongated and had straight or irregular cristae. In ST 3 cells, we observed some swollen mitochondria of a larger than normal diameter, with a light matrix and rare cristae. From ST 3 to ST 5 swollen mitochondria became increasingly abundant (arrow in one ST4 cell).

## Discussion

We aimed to quantify the changes in water and element/ion contents during each step of apoptosis. We began by characterizing the changes in the nucleus occurring during mitochondrial depolarization in living cells. We then used chromatin fluorescence on ultrathin cryo-sections to identify each stage of apoptosis. We then carried out correlative cryo-imaging [9, 10], in a targeted ultrastructural cryo-analysis of the water and element/ion contents of the main compartments of apoptotic cells.

The first part of our study on living cells demonstrated an increase in nuclear volume preceding ST 1, consistent with the reported swelling of the whole cell before entry into apoptosis [21]. We identified ST 1 as the onset of apoptosis, on the basis of mitochondrial depolarization. We confirmed that the latter occurred suddenly and was completed within 10 to 15 minutes and that this process was caspase 3-independent [18, 22, 23]. During ST1, the swollen nucleus contained a large amount of Cc but no marginated condensed chromatin. Cell shrinkage (i.e. AVD), in response to caspase-3 activation and the cleavage of its substrates [19], began once mitochondrial depolarization was complete. The characteristic reorganization of chromatin was used to identify ST 2 to ST 5 and occurred at the same time as a steady decrease in the volume of the nucleus to 40% of its initial volume.

Previous studies determining cell volume and water content by light microscopy [3, 7] during apoptosis obtained conflicting results [8]. Our direct quantification of dry mass and water content through the application of dark-field imaging to ultrathin cryosections [9] showed that water content was 10 to 15% higher in all compartments of apoptotic cells than in control cells. However, as the water content of cells not engaged in apoptosis (ST 0) was similar to that of apoptotic cells, an increase in water content is clearly not sufficient to induce apoptosis. As previously suggested [1, 2], apoptosis is not regulated by the change in cell volume due to a loss of water, but by the flux of ions. We investigated changes in the concentration of elements and ions within the organelles during the various stages of apoptosis, by carrying out EDX analysis on ultrathin cryo-sections [9, 10]. Unlike methods based on the use of fluorescent markers [24], EDX analysis can be used for the simultaneous accurate identification and quantification of numerous elements/ions in regions of interest (ROI) [25–27]. Our cryo-correlative approach has two additional advantages. It clearly identifies nuclear compartments and physiological concentrations of ions/elements can be calculated in mmol/L.

**Fig 7:**
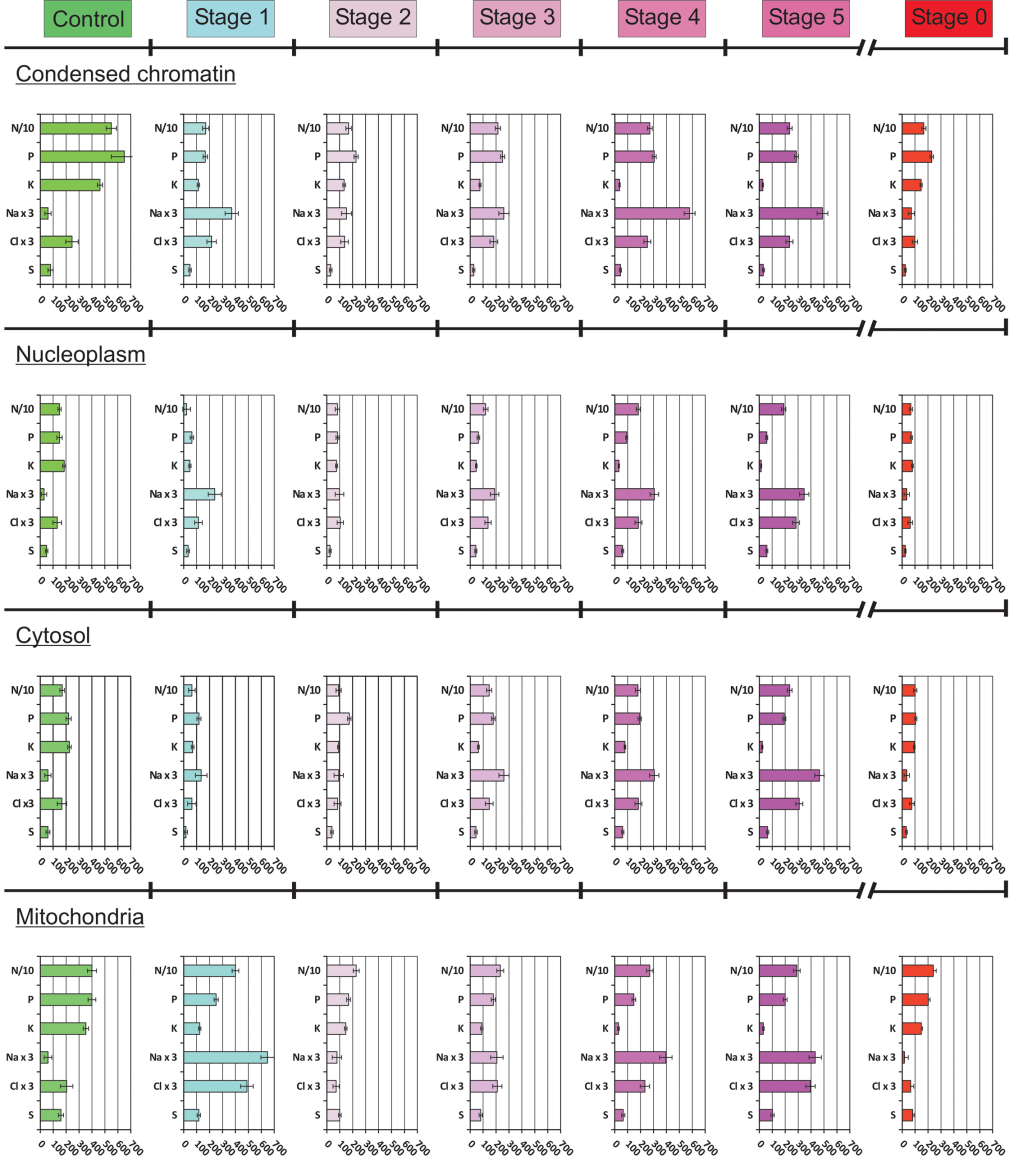
“Elemental descriptors” gathering concentration of all the elements/ions considered, for each compartment (condensed chromatin, nucleoplasm, cytosol and mitochondria) in control cells, in cells during each stage of apoptosis and during stage 0. In the histograms, concentration of nitrogen was divided by 10 (N/10), whereas the concentration of sodium and chloride was multiplied by 3 (Na x 3 and Cl x 3).

In order to easily compare the changes in the concentration of all ions/elements within each cell compartment during the steps of apoptosis, we compiled the data and plotted them on histograms to obtain an “element descriptor” of the compartment concerned (Fig 7). The use of the same scale on all these histograms allows for the easy visualisation of changes: i) during the different steps of apoptosis for one compartment and ii) during a given step of apoptosis for all compartments.The status of the elements and ions within each compartment was used to define three main periods: ST 1, ST 2 and ST 3 to ST 5. During ST 1, mitochondrial [Na^+^] and [Cl^-^] increased considerably, to 220 mmol/L and 160 mmol/L, respectively, whereas [K^+^] decreased to 100 mmol/L. Simultaneously, [Na^+^] reached 50 mmol/L in the cytosol and 80 to 120 mmol/L in the nucleoplasm and chromatin, whereas [Cl^-^] decreased to 20 mmol/L in the cytosol but remained constant in the nucleus (50 to 80 mmol/L). During ST 2 (the first step of AVD [1, 2]), mitochondrial [Na^+^] and [Cl^-^] decreased to levels similar to those in the cytosol (50 and 20 mmol/L, respectively) and reached 50 mmol/L in the nucleus. From ST 3 to ST 5 (second step of AVD [1, 2]), [K^+^] decreased steadily to 20 mmol/L in all compartments. Conversely, [Na^+^] and [Cl^-^] increased steadily, from 100 to 150 mmol/L and from 80 to 140 mmol/L respectively.

Our findings for mitochondria are consistent with those obtained by Yang’s group using fluorescence imaging of Na^+^ on cardiomyocytes at the onset of apoptosis [28, 29]. They detected an increase in mitochondrial Na^+^ concentration, with a restoration to normal levels, due to TRPM2 channel activation and reverse-mode activation of the Na^+^/Ca^2+^ exchanger (rNCX). They showed that mitochondrial Na^+^ (mt[Na^+^]) overload was sufficient to induce apoptosis by activating caspase-3. Our results are consistent with these data, as we found that mt[Na^+^] increased before caspase-3 activation. We found that mt[Na^+^] and mt[Cl^-^] reached very high levels, much greater than ever reported before. As cytosolic [Cl^-^] varied slightly, it is possible that mitochondrial Cl^-^ channels were involved in the increase in mt[Cl^-^] [30]. We observed no change in mitochondrial morphology during ST 1, as previously shown [31], or during ST 2. This suggests that mitochondrial volume is strongly regulated during the onset of apoptosis and early stages of shrinking. Conversely, the progressive swelling of the mitochondria, beginning at ST 3, when mt[Na^+^] and mt[Cl^-^] re-increased may reflect the action of caspases in influx of water via aquaporins [31, 32].

Caspase 3 was functional in ST 2 to ST 5, but not functional in control cells or in ST 1 and ST 0. We were therefore able to correlate the ion status with caspase 3 activity in the cytosol and nucleoplasm. Caspase 3 was active: i) when[K^+^] was below 100 mmol/L, ii) when [Na^+^] and [Cl^-^] were each above 25 mmol/L and iii) when the ratio of [Na^+^] /[Cl^-^] was between 1 and 2. These data extend the findings of previous studies reporting that apoptosis was not activated exclusively by a decrease in [K^+^] [33, 34] but was instead dependant on changes in ion status [1, 2, 35], such as an increase in [Na^+^] or in both [Na^+^] and [Cl^-^] [36, 37].

In the nuclei of ST 1 cells, [K^+^] decreased from 460 mmol/L to 100 mmol/L in condensed chromatin whereas [Na^+^] increased from 20 to 130 mmol/L. During subsequent stages, the increase in chromatin compaction was accompanied by a steady decrease in [K^+^] to 20 mmol/L and an increase in [Na^+^] to 150 mmol/L in ST 5. [Cl^-^] and [Mg^2+^] simultaneously reached 80 mmol/L and 15 mmol/L, respectively. These *in vivo* ionic conditions are consistent with recent reports that chromatin compaction *in vitro*, in the presence of Mg^2+^, is limited by a high [K^+^] but promoted by a high [Na^+^] [38]. We also confirmed the presence of Cc in the nuclear compartments of apoptotic cells [20] except for condensed chromatin. This finding is consistent with the reported interactions between Cc and nuclear proteins involved in RNA synthesis and the DNA damage response [39] located outside the condensed chromatin [40]. When [Na^+^] is high, as during ST1, 20% of the nucleosomes dissociate, releasing free DNA [41]. Cc is a highly positively charged protein [42]. It may therefore interact with uncondensed DNA. This interaction might cause the partial unfolding and phosphorylation of Cc, as observed during its interaction with cardiolipin[42, 43] and its conversion into a peroxidase that cleaves DNA [44]. DNA cleavage may be responsible for the lower level of H2B-GFP fluorescence outside the condensed chromatin during ST 2. This finding is consistent with the cleavage of high-molecular weight DNA observed before caspase-activated DNase activity [45, 46]. Moreover, the lack of Cc immunolabeling observed with a mAb unable to detect the denatured form of Cc was consistent with the presence of a putative unfolded form of Cc at ST 2 [47]. The unmodified Cc became detectable again with this antibody, in both the cytoplasm and nuclei of cells during ST 3 to ST 5.

We also characterized cells in ST 0, which had not engage in apoptosis after seven hours of AMD treatment and, for some of them, were able to reengage in proliferation three days after AMD withdrawal (S5 Movie). Such cells, emerging after the use of apoptotic inducers [13, 48] constitute a population of drug-tolerant cells [49]. In our study, the presence of segregated nucleoli showing a very dense cap of fibrillar component due to rRNA synthesis inhibition by AMD [50, 51] demonstrated that this compound had entered the ST0 cells. Our elemental analysis identified these cells as very similar to stressed cells in which we specifically inhibited rRNA synthesis with a low dose of AMD [10]. The compartments of ST 0 cells were characterised by water and K^+^ contents similar to those of ST1 cells, but much lower Na^+^ and Cl^-^ contents.

## Conclusion

In this study, we simultaneously analysed changes in the water and element/ion contents of cells during all steps of apoptosis with unprecedented precision. We found that cells treated with a high dose of AMD had a lower K^+^ content and a hydration index twice that of control cells, in all their compartments. Moreover, cells engaged in apoptosis (ST 1 to ST 5 cells) had higher Na^+^ and Cl^-^ contents than drug-tolerant cells. This is consistent with previous reports that an increase in sodium chloride concentration by artificial transporters is sufficient to induce mitochondrial outer membrane permeabilization (MOMP) and caspase activation [37]. We demonstrated a large increase of mitochondrial Na^+^ and Cl^-^ contents at the onset of apoptosis. We also observed large differences in Na^+^, Cl^-^ and K^+^ contents that were characteristic of the various stages and cell compartments. All these findings suggest that cytosolic and mitochondrial Na^+^, Cl^-^ and K^+^ fluxes are early events that might “truly push cells beyond the point-of-no-return separating life and death” [1, 2, 14].

## Supporting Information

S1 Fig Lookup scale used in Fig 4. To easily identify the different levels of fluorescence imaged in the ultrathin cryosection, we applied a rainbow lookup table (LUT) shown here. This was used to: i) merge fluorescence image on STEM image and ii) draw regions of interest in which STEM imaging was performed for quantitation of water and for ions identification and quantitation by energy dispersive X-ray spectrometry.
(TIF)

S2 Fig Simultaneous 3D localization of activated caspase-3 (actCASP3), cleaved PARP (clePARP) and H2B-GFP, demonstrating that ST 2 to ST 5 cells have entered apoptosis. Anti-activated caspase-3 and anti-cleaved PARP antibodies were used to label fixed HeLa cells stably expressing H2B-GFP after the induction of apoptosis by incubation with 500 ng/mL AMD for 7 h 15 minutes. Four images are shown for the same cell at a given stage. The first image is an optical section passing through the middle of the nucleus and showing a merge of actCASP3 (blue), clePARP (red) and H2B-GFP (green) labeling. The second image is a 3D surface rendering of actCASP3 (blue) and clePARP (red) labeling. The third image is a 3D surface rendering of actCASP3 (blue), clePARP (red) and H2B-GFP (green) labeling. The final image is a simultaneous 3D transparent volume rendering of actCASP3 (blue), clePARP (red) and H2B-GFP (green) labeling. The scale bar represents 10 μm.
(TIF)

S3 Fig Ultrastructural morphology studies, demonstrating the presence of swollen mitochondria in ST 3, ST 4 and ST 5 cells. We investigated the shape of mitochondria and the structure of their cristae in ultrathin cryo sections of directly cryo fixed HeLa H2B-GFP cells. In control (ctrl), ST 0 cells, ST 1 and ST 2 cells, mitochondria were elongated and had straight or slightly curved cristae. In ST 3 cells, rare swollen mitochondria were observed, with a larger than normal diameter, a light matrix and rare cristae. These swollen mitochondria became increasingly abundant during ST 4 and ST 5 (see arrow on ST4 image for example). The scale bar represents 0.5 μm.
(TIF)

S1 Movie Simultaneous imaging of mitochondrial potential and of nuclear modifications studied by time-lapse confocal imaging after the induction of apoptosis by 500 ng/mL AMD. HeLa cells stably expressing H2B-GFP were stained with TMRE to study mitochondrial polarization. Simultaneous time-lapse confocal imaging of TMRE and H2B-GFP was performed by two-photon excitation, every 5 minutes for 7 h and 15 minutes after the induction of apoptosis by the addition of 500 ng/mL AMD. *Z*-stacks of 85 optical sections were processed to obtain 3D images (surface rendering for chromatin and volume rendering for mitochondrial polarization), which were assembled to produce movies demonstrating the successive steps of 3D chromatin reorganization and changes in mitochondrial potential (based on the green fluorescence of H2B-GFP and red fluorescence of TMRE respectively). In the field of view shown in this movie, we observed 35 cells containing H2B-GFP. Twelve cells entering apoptosis during the period of 7 hours and 15 minutes were numbered 1 to 12. In these cells, depolarization of mitochondria was initiated at different times as demonstrated by the observation of TMRE red signal reorganization. Mitochondrial polarization disappeared after: three hours and 45 minutes (cell # 1), four hours and 35 minutes (cell # 2), five hours and 10 minutes (cells # 3 and 4), six hours (cells # 5 and 6), six hours and 5 minutes (cell # 7), six hours and 15 minutes (cells # 8 and 9), six hours and 20 minutes (cell # 10) and six hours and 30 minutes (cells # 11 and 12).
(MOV)

S2 Movie DIC imaging of cells studied by time-lapse confocal imaging after the induction of apoptosis by 500 ng/mL AMD. Same experiment and same field of view as shown on S1 Movie. During GFP and TMRE fluorescence imaging, Z-stacks of DIC images were acquired. We choosed one characteristic optical section from each z-stack and we used it to build a movie showing the structural modifications of the cells during the steps of apoptosis.
(MOV)

S3 Movie Simultaneous imaging of mitochondrial potential and of nuclear modifications studied by time-lapse confocal imaging after the induction of apoptosis by 500 ng/mL AMD. Same experiment as the one shown in S1 Movie, but the cells numbered 2 and 9 are shown at a higher zoom. The cell numbered 9 (on the right-hand side) was previously shown in details on Fig 1. Mitochondrial polarization disappeared at four hours and 35 minutes (cell # 2) and at six hours and 15 minutes (cell # 9).
(MOV)

S4 Movie Simultaneous imaging of mitochondrial potential and of nuclear modifications studied by time-lapse confocal imaging after the induction of apoptosis by 500 ng/mL AMD. HeLa cells stably expressing H2B-GFP were stained with TMRE to study mitochondrial polarization. Simultaneous time-lapse confocal imaging of TMRE and H2B-GFP was performed by two-photon excitation, every 7 minutes for 15 h and 17 minutes after the induction of apoptosis by the addition of 500 ng/mL AMD. *Z*-stacks of 85 optical sections were processed to obtain 3D images (surface rendering for chromatin and volume rendering for mitochondrial polarization), which were assembled to produce movies demonstrating the successive steps of 3D chromatin reorganization and changes in mitochondrial potential (based on the green fluorescence of H2B-GFP and red fluorescence of TMRE respectively). In the field of view shown in this movie, we observed 32 cells containing H2B-GFP. Depolarization of mitochondria, as demonstrated by the observation of TMRE red signal reorganization, occurred at three hours and 30 minutes in the first cell and at fourteen hours in the last cell.
(MOV)

S5 Movie Long-term imaging of nuclear modifications induced during 500 ng/mL AMD treatment and subsequent recovery after AMD washing out studied by time-lapse confocal imaging of HeLa cells stably expressing H2B-GFP. Time-lapse confocal imaging of H2B-GFP was performed by two-photon excitation, every 5 minutes. *Z*-stacks of 85 optical sections were processed to obtain 3D images of chromatin and were assembled to produce one movie demonstrating the successive steps of 3D chromatin reorganization. Cells in exactly the same area were imaged during successive periods and conditions. During the first period of time (0 to 7h), cells were maintained in contact with AMD to induce apoptosis. At the end of this period, 7 cells (on a total of 34 cells) were engaged in apoptosis. Cells were then rinsed several times in PBS and fresh culture medium to wash out AMD. During the second period (7h45 to 22h 16 minutes), cells were maintained in culture medium without AMD and the same area was imaged. At the end of this period, 22 cells were engaged in apoptosis and 12 were not engaged in apoptosis (unaffected cells or stage 0 cells). Culture medium without AMD was changed each day during 3 days and then cells in the same field of view were imaged during 6 hours and 25 minutes. During this period, 10 cells were imaged: none engaged in apoptosis and five entered mitosis.
(MOV)

S6 Movie Simultaneous 3D localization of cytochrome-*c* (Cc) and H2B-GFP showing Cc redistribution during specific stages of apoptosis. Anti-cytochrome-*c* antibody binding was imaged on fixed HeLa cells stably expressing H2B-GFP after the induction of apoptosis by 500 ng/mL AMD, for 7h and 15 minutes. For each stage of apoptosis and stage 0, Z-stacks were processed for a simultaneous 3D transparent volume rendering of Cc (red) and H2B-GFP (green) which was then fully rotated. The six cells (ST1 to ST5 and ST0) are shown in the same window to improve comparison of Cc and H2B-GFP localization.
(MOV)

S7 Movie Simultaneous 3D localization of activated caspase-3 (actCASP3), cleaved PARP (clePARP) and H2B-GFP, demonstrating that ST 2 to ST 5 cells have entered apoptosis. Anti-activated caspase-3 and anti-cleaved PARP antibodies were used to label fixed HeLa cells stably expressing H2B-GFP after the induction of apoptosis by incubation with 500 ng/mL AMD for 7 h 15 minutes. For each stage of apoptosis and stage 0, Z-stacks were processed for a simultaneous 3D transparent volume rendering of actCASP3 (blue), clePARP (red) and H2B-GFP (green) which was then fully rotated. The six cells (ST1 to ST5 and ST0) are shown in the same window to improve comparison of actCASP3, clePARP and H2B-GFP localization.
(MOV)

S1 Table Data used for quantitation of the nuclear volume (in % of nuclear volume at time 0) and of TMRE intensity (in % of TMRE intensity at time 0.91 H) for some cells shown on S1 and S2 Movies (cell # 2, 5, 7, 9 and 11 and one unaffected cell (stage 0 cell)) and on Fig 1 (cell # 9). (XLSX)

S2 Table Data obtained by Scanning Transmission Electron Microscopy imaging to quantify water percentage in cell compartments (condensed chromatin, nucleoplasm, cytosol and mitochondria) in control cells and in cells in various stages of apoptosis (stage 1, stage 2, stage 3, stage 4, stage 5) and cells unaffected by actinomycin D treatment (cells in stage 0). These data were shown in Fig 4.
(XLSX)

S3 Table Data obtained by Scanning Transmission Electron Microscopy (energy dispersive X-ray spectrometry) to quantify concentration of elements/ions (N, P, K^+^, Na^+^, Cl^-^, S and Mg^2+^) in cell compartments (condensed chromatin, nucleoplasm, cytosol and mitochondria) in control cells and in cells in various stages of apoptosis (stage 1, stage 2, stage 3, stage 4, stage 5) and cells unaffected by actinomycin D treatment (cells in stage 0). These data were shown in Figs 5, 6 and 7.
(XLSX)
