# Supplementary figures and images for: Stage-Specific Changes in the Water, Na+, Cl- and K+ Contents of Organelles during Apoptosis, Demonstrated by a Targeted Cryo Correlative Analytical Approach
Source: PLoS One. 2016 Feb 11;11(2):e0148727. doi: 10.1371/journal.pone.0148727 (PMC4807926; doi:10.1371/journal.pone.0148727)

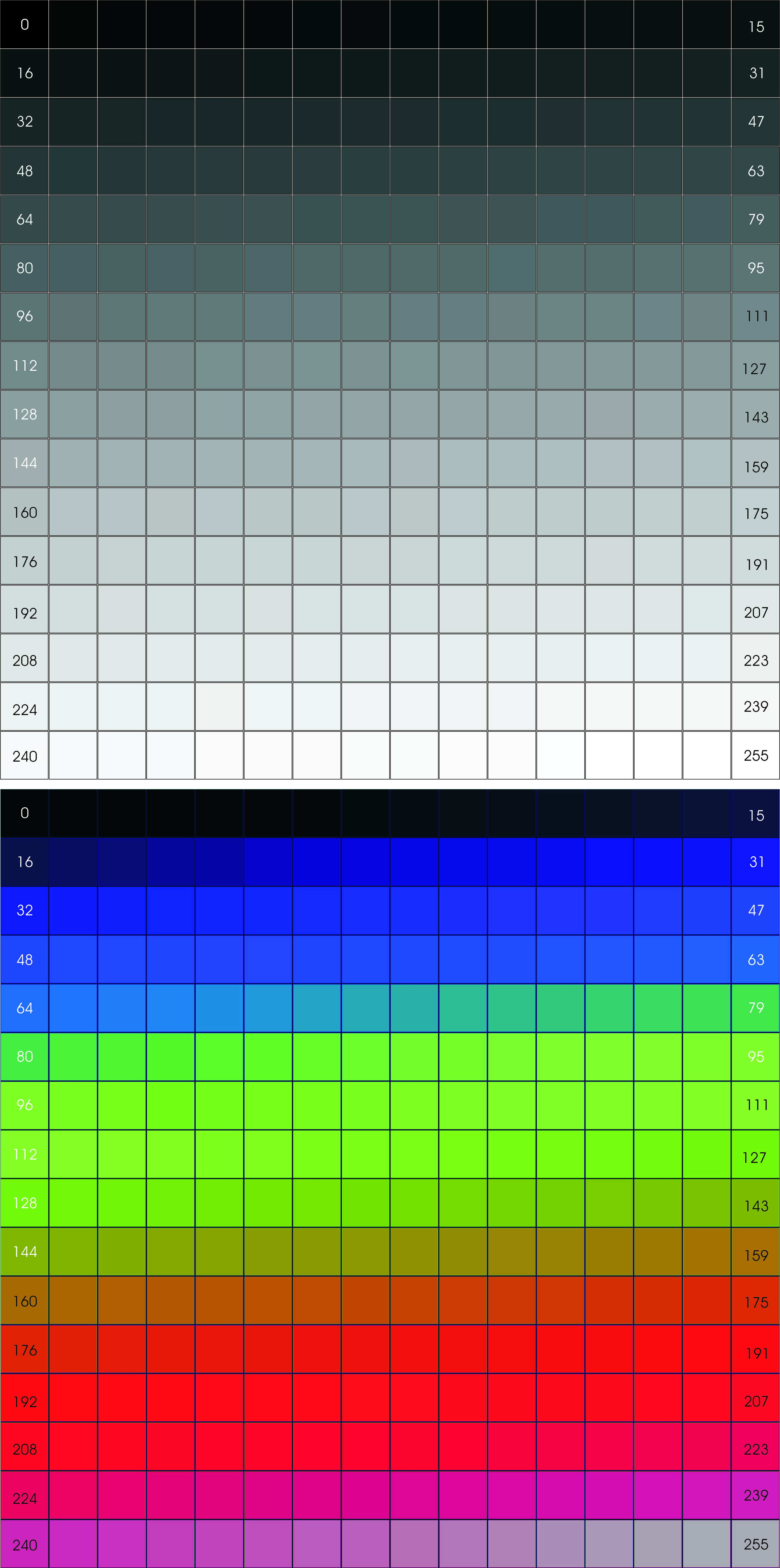

Supplement: S1 Fig — To easily identify the different levels of fluorescence imaged in the ultrathin cryosection, we applied a rainbow lookup table (LUT) shown here. This was used to: i) merge fluorescence image on STEM image and ii) draw regions of interest in which STEM imaging was performed for quantitation of water and for ions identification and quantitation by energy dispersive X-ray spectrometry. (TIF) [file pone.0148727.s001.tif]

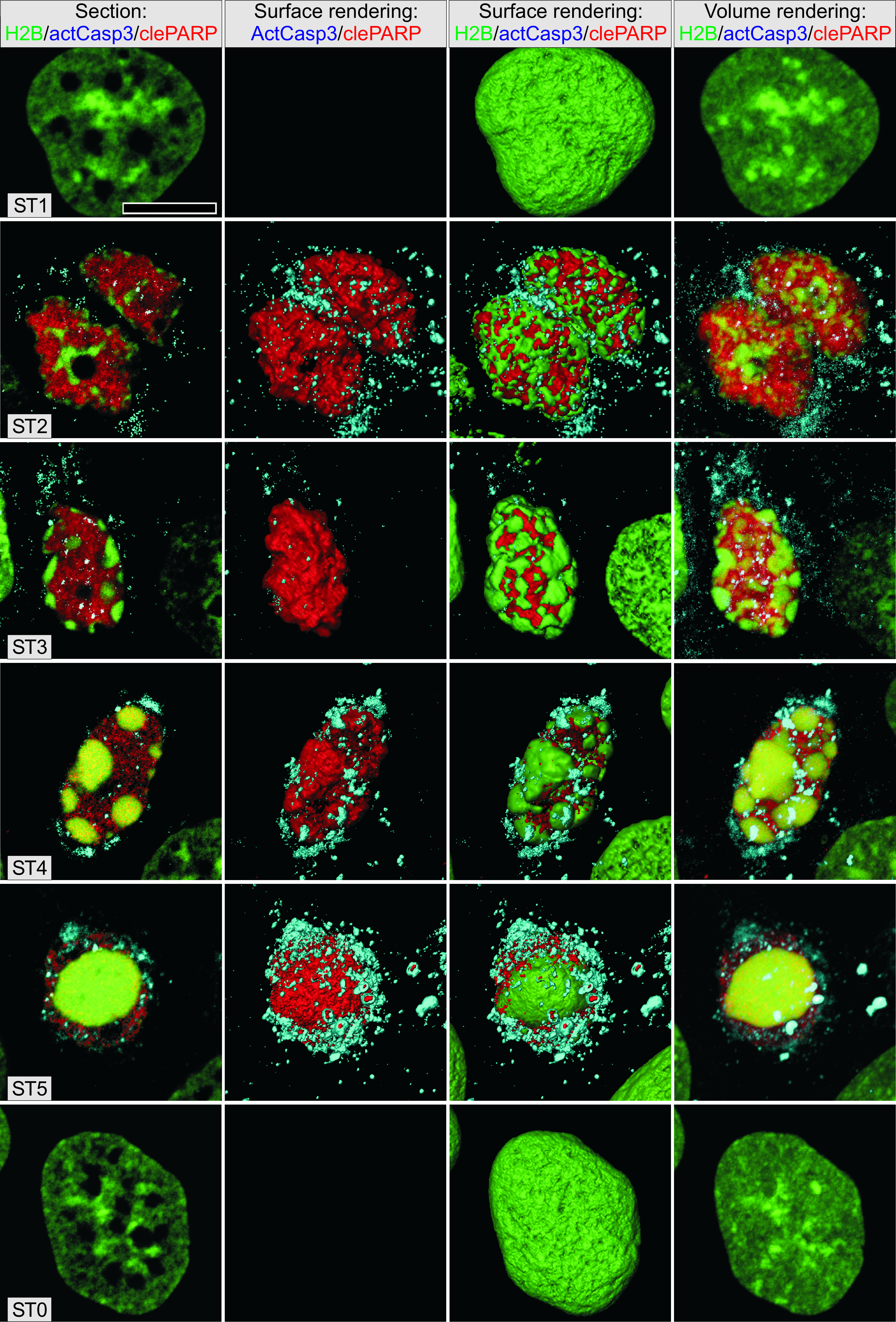

Supplement: S2 Fig — Anti-activated caspase-3 and anti-cleaved PARP antibodies were used to label fixed HeLa cells stably expressing H2B-GFP after the induction of apoptosis by incubation with 500 ng/mL AMD for 7 h 15 minutes. Four images are shown for the same cell at a given stage. The first image is an optical section passing through the middle of the nucleus and showing a merge of actCASP3 (blue), clePARP (red) and H2B-GFP (green) labeling. The second image is a 3D surface rendering of actCASP3 (blue) and clePARP (red) labeling. The third image is a 3D surface rendering of actCASP3 (blue), clePARP (red) and H2B-GFP (green) labeling. The final image is a simultaneous 3D transparent volume rendering of actCASP3 (blue), clePARP (red) and H2B-GFP (green) labeling. The scale bar represents 10 μm. (TIF) [file pone.0148727.s002.tif]

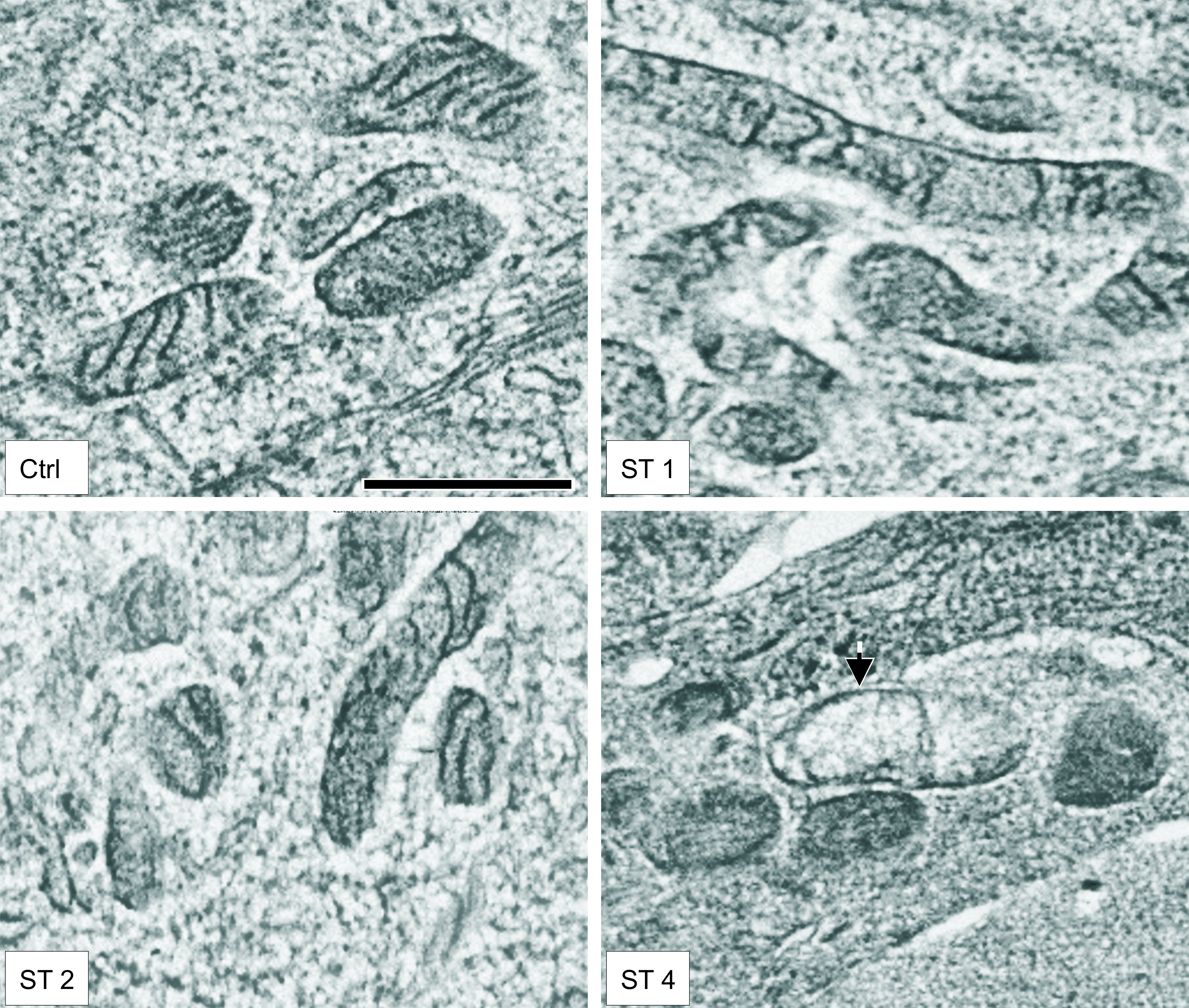

Supplement: S3 Fig — We investigated the shape of mitochondria and the structure of their cristae in ultrathin cryo sections of directly cryo fixed HeLa H2B-GFP cells. In control (ctrl), ST 0 cells, ST 1 and ST 2 cells, mitochondria were elongated and had straight or slightly curved cristae. In ST 3 cells, rare swollen mitochondria were observed, with a larger than normal diameter, a light matrix and rare cristae. These swollen mitochondria became increasingly abundant during ST 4 and ST 5 (see arrow on ST4 image for example). The scale bar represents 0.5 μm. (TIF) [file pone.0148727.s003.tif]
